# Roles of Deubiquitinases OTUD3 and OTUD5 in Inflammatory Bowel Diseases

**DOI:** 10.3390/ijms26209924

**Published:** 2025-10-12

**Authors:** Tomohiro Watanabe, Masatoshi Kudo

**Affiliations:** Department of Gastroenterology and Hepatology, Kindai University Faculty of Medicine, 377-2 Ohno-Higashi, Osaka-Sayama 589-8511, Japan

**Keywords:** inflammatory bowel disease, NOD2, STING, TLR9, TRAF3, type I interferon

## Abstract

Excessive production of type I interferons (IFNs) underlies the immunopathogenesis of autoimmune disorders, including systemic lupus erythematosus and autoimmune pancreatitis. Whether type I IFNs play pathogenic or protective roles in the development of inflammatory bowel diseases (IBD) has been a matter of debate. The production of type I IFNs is tightly regulated by the conjugation and removal of polyubiquitin chains on or from intracellular signaling molecules. OTU deubiquitinases 3 (OTUD3) and 5 (OTUD5) are enzymes that cleave various polyubiquitin chains from target proteins. OTUD3 and OTUD5 deubiquitinate key critical intracellular molecules of the type I IFN signaling pathways, stimulator of interferon genes (STING), and TNF receptor-associated factor 3 (TRAF3), respectively, and thus regulate the production of type I IFNs by innate immune cells. Recent studies provided evidence that the impaired function of OTUD3 and OTUD5 increases susceptibility to human and experimental IBD owing to the excessive production of type I IFNs caused by the activation of STING and TRAF3, respectively. Collectively, OTUD3 and OTUD5 play protective rather than pathogenic roles in the development of IBD through the negative regulation of type I IFN-mediated signaling pathways. In this review article, we discuss the association between the development of IBD and impaired function of OTUD3 or OTUD5 by focusing on their deubiquitinase activity and type I IFN responses.

## 1. Introduction

Inflammatory bowel diseases (IBDs) characterized by recurrent and persistent gastrointestinal (GI) tract inflammation are mainly categorized into Crohn’s disease (CD) and ulcerative colitis (UC) [1,2]. Excessive production of pro-inflammatory cytokines by gut macrophages and dendritic cells (DCs) upon the recognition of intestinal bacteria by pattern recognition receptors (PRRs) underlies the development of CD and UC [1,2,3]. Strong support for this concept comes from the remarkable success of biologics targeting prototypical colitogenic cytokines, such as IL-12, IL-23, and TNF-α, in the treatment of patients with IBD [1,2].

Type I interferons (IFNs), which are produced by macrophages, DCs, and epithelial cells upon sensing invading pathogens by PRRs, play a central role in antimicrobial host defense [4]. Although the activation of the type I IFN signaling pathway is beneficial for early host defense against microbial infections, excessive and persistent production of type I IFNs triggers the development of autoimmune disorders. Autoimmune pancreatitis and systemic lupus erythematosus are driven by type I IFNs produced by plasmacytoid DCs (pDCs) [5,6,7]. No definitive conclusions have been reached regarding the role of type I IFNs in the development of IBD. The recognition of bacterial nucleic acids by Toll-like receptor 9 (TLR9) induces robust production of type I IFNs by macrophages and DCs [6,7]. Activation of TLR9 by subcutaneous injection of agonistic CpG-containing double-stranded DNA (dsDNA) prevents the development of dextran sodium sulfate (DSS)-induced colitis in a type I IFN-dependent manner when TLR9 agonists were administered before colitis induction [8,9]. Such preventive effects of type I IFNs released following TLR9 activation on DSS-induced colitis are associated with intestinal barrier restoration [10]. In contrast, when TLR9 was activated by its agonists during the induction of colitis, the activation of type I IFN signaling exacerbated DSS-induced colitis by promoting T helper type 1 responses [11,12,13]. Exacerbation of experimental colitis by TLR9-mediated type I IFN responses is consistent with enhanced expression of type I IFN signature genes in the colonic mucosa of patients with IBD [14,15].

Production of type I IFNs is regulated by the conjugation and removal of polyubiquitin chains on or from signaling molecules [16]. OTU deubiquitinases 3 (OTUD3) and 5 (OTUD5) are regulators of type I IFN responses [17,18]. Stimulator of interferon genes (STING) and TNF receptor-associated factor 3 (TRAF3) are key signaling molecules located downstream of PRRs involved in the production of type I IFNs. OTUD3 and OTUD5 remove polyubiquitin chains from STING and TRAF3, thereby preventing excessive production of type I IFNs [17,18]. Recent studies have provided evidence that enhanced activation of type I IFN signaling pathways due to the impaired function of OTUD3 and OTUD5 increases susceptibility to human and experimental IBD. Here, we discuss the association between the regulation of type I IFN responses by OTUD3 and OTUD5 and the development of IBD.

## 2. Deubiquitination by OTUD3 and OTUD5

Ubiquitination is a post-translational process mediated by ligases that conjugate polyubiquitin chains to target proteins [19,20,21]. Polyubiquitin chains are classified into eight types according to the amino acids that provide attachment sites for the elongation of ubiquitin molecules, thereby forming polymeric chains. Seven lysine residues of ubiquitin (K6, K11, K27, K29, K33, K48, and K63) and its N-terminal methionine (M1) have been identified as the attachment sites [22,23]. K11- or K48-linked polyubiquitination of target proteins induces proteasomal degradation, whereas K27-, K63-, or M1-linked polyubiquitination triggers nuclear translocation of nuclear factor-κB (NF-κB) subunits and interferon regulatory factors (IRFs) through the formation of signaling scaffolds decorated with polyubiquitin chains [22,23]. Polyubiquitination of signaling molecules associated with NF-κB and IRF signaling pathways regulates cytokine responses because nuclear translocation of NF-κB subunits and IRFs is required for the production of cytokines [3].

Polyubiquitination by ligases is antagonized by deubiquitinases, which remove the polyubiquitin chains [19,20,21]. The balance between ubiquitination and deubiquitination is tightly regulated, and its dysregulation is associated with many diseases, including cancer and autoimmunity [19,20,21]. OTUD3 and OTUD5, the latter of which is also known as deubiquitinating enzyme A, belong to the OTUD subfamily [19,20]. OTUD3 cleaves K27-, K48-, and K63-linked polyubiquitin chains, whereas polyubiquitin chains removed by OTUD5 are limited to K48- or K63-linked ones [19,20]. Deubiquitination of targeted proteins by OTUD3 and OTUD5 controls innate immune responses, particularly type I IFN responses, mediated by the activation of PRRs, including TLRs, retinoic acid-inducible gene-I (RIG-I)-like receptors (RLRs), and cyclic GMP-AMP synthase (cGAS) [19,20].

## 3. Type I IFN Signaling Pathways

**Figure 1:**
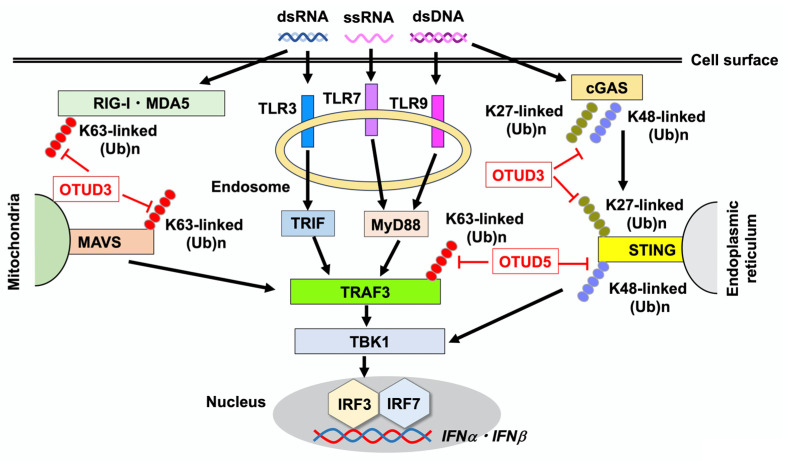
Regulation of type I IFN responses by OTUD3 and OTUD5. Toll-like receptors (TLRs), retinoic acid-inducible gene-I (RIG-I)-like receptors (RLRs), and cyclic GMP-AMP synthase (cGAS) are pattern recognition receptors that detect double-stranded DNA (dsDNA), double-stranded RNA (dsRNA), and single-stranded RNA (ssRNA) derived from viruses and bacteria. Endosomal TLR3, TLR7, and TLR9 detect dsRNA, ssRNA, and dsDNA, respectively. Intracellular RIG-I and melanoma differentiation-associated protein 5 (MDA5) sense dsRNA, whereas cGAS detects dsDNA. Recognition of nucleic acids by TLRs and RLRs leads to Lys (K)-linked polyubiquitination on TNF receptor-associated factor 3 (TRAF3), which results in the transcription of *IFNα* and *IFNβ* through the nuclear translocation of interferon regulatory factor 3 (IRF3) and IRF7 following activation of TANK-binding kinase 1 (TBK1). TIR-domain-containing adapter molecule inducing interferon-β (TRIF), myeloid differentiation factor 88 (MyD88), and mitochondrial antiviral-signaling protein (MAVS) are adaptor molecules for TLR3, TLR7/9, and RLRs, respectively. Stimulator of interferon genes (STING), which is an adaptor molecule for cGAS, induces the transcription of *IFNα* and *IFNβ* through the nuclear translocation of IRF3 and IRF7. Defective cleavage of the K27-linked polyubiquitination of STING is associated with the immunopathogenesis of ulcerative colitis in the presence of *Otud3* single nucleotide polymorphism. OTUD3 also cleaves K63-linked polyubiquitin chains on RIG-I, MDA, and MAVS to downregulate type I IFN responses. Removal of K27- or K48-linked polyubiquitination of cGAS, which stabilizes the protein, is mediated by OTUD3. OTUD5 mediates the removal of K63-linked polyubiquitination of TRAF3 to downregulate TLR- and RLR-mediated type I IFN responses. K48-linked polyubiquitination of STING is cleaved by OTUD5 to upregulate the cGAS-mediated type I IFN response.

Production of type I IFNs is triggered by the sensing of microbial and host nucleic acids by PRRs [16,24,25,26]. TLRs, RLRs, and cGAS are the major PRRs that detect microbial and host endogenous DNA/RNA and initiate strong type I IFN responses [16,24,25,26]. TLR3, TLR7, and TLR9 are endosomal TLRs that induce robust production of type I IFNs by macrophages and DCs upon sensing microbial nucleic acids (Figure 1) [27,28]. TLR3 and TLR7 mainly recognize viral double-stranded RNA (dsRNA) and single-stranded RNA (ssRNA), respectively [27,28]. TLR9 detects CpG-containing dsDNA derived from bacteria. Nucleic acid sensing by TLR7 and TLR9 activates adaptor molecule myeloid differentiation factor 88 (MyD88), whereas sensing by TLR3 activates TIR-domain-containing adapter molecule inducing interferon-β (TRIF) [16,27,28]. TLR3, TLR7, and TLR9 share TRAF3 as a crucial adaptor molecule and binding of TRIF or MyD88 to TRAF3 transactivates IRF3 and IRF7 to initiate the transcription of type I IFN genes [16,27,28].

RLRs are composed of RIG-I, melanoma differentiation-associated protein 5 (MDA5), and laboratory of genetics and physiology 2 protein (Figure 1) [26,27,28]. RLRs function as viral RNA sensors and play critical roles in antiviral host defense by inducing type I IFN responses [26,27,28]. Detection of viral RNA by RIG-I and MDA5 activates mitochondrial antiviral-signaling protein (MAVS), followed by the nuclear translocation of IRF3 and IRF7 to induce optimal production of type I IFNs [26,27,28]. Similar to TLR3, TLR7, and TLR9, TRAF3 functions as an adaptor molecule connecting MAVS and IRFs in RLR-mediated signaling pathways [26,27,28].

cGAS senses cytosolic dsDNA derived from microorganisms and the host, being a crucial component of the innate immune system in addition to TLRs and RLRs [25,29] (Figure 1). Upon sensing of dsDNA, cGAS synthesizes the cyclic second messenger 2′,3′-cyclic GMP-AMP (cGAMP) that engages STING to induce production of type I IFNs through the nuclear translocation of IRF3 [25,29]. Collectively, the TLR3/TLR7/TLR9-TRIF/MyD88-TRAF3, RIG-I/MDA5-MAVS, and cGAS-STING cascades constitute three major signaling pathways that lead to the production of type I IFNs upon sensing microbial and host nucleic acids. TANK-binding kinase 1 (TBK1), an intracellular protein downstream of TLRs, RLRs, and cGAS-STING is an immediate upstream molecule of IRFs.

## 4. Regulation of Type I IFN Responses by OTUD3 and OTUD5

OTUD3 and OTUD5 regulate type I IFN responses through their deubiquitinase activities. OTUD3 cleaves K63-linked polyubiquitin chains from RIG-I and MDA5, thereby suppressing type I IFN responses upon infection with RNA virus [30] (Figure 1). Production of IFN-β and C-X-C motif chemokine ligand 10 (CXCL10), a prototypical type I IFN-dependent chemokine, was markedly higher in bone marrow (BM)-derived DCs and murine lung fibroblasts isolated from OTUD3-deficient mice than in those from wild-type mice upon exposure to an RNA virus [30]. In addition, OTUD3-deficient mice were more resistant to lethal encephalomyocarditis virus (RNA virus) infection than wild-type mice owing to the enhanced activation of type I IFN signaling pathways. MAVS is a crucial downstream molecule in the RLR-mediated signaling pathway. The K63-linked polyubiquitination of RIG-I, MDA5, and MAVS is removed by OTUD3 [30,31]. Thus, OTUD3 functions as a negative regulator of RLR-mediated type I IFN responses by removing the K63-linked polyubiquitin chains from RIG-I, MDA5, and MAVS. Deubiquitinase activity of OTUD3 is controlled by acetylation during RNA virus infection [31]. In response to a viral infection, acetylated Lys129 of OTUD3 is removed by SIRT1, and the catalytic activity of this deubiquitinase decreases, allowing induction of antiviral type I IFN responses.

In contrast to the RLR signaling pathways, OTUD3 promotes cGAS-STING-mediated type I IFN responses. OTUD3 hydrolyzes K27- and K48-linked polyubiquitination of cGAS enhancing its stability and enzymatic activity [30,32]. OTUD3-deficient mice were more sensitive to lethal herpes simplex virus (DNA virus) infection than wild-type mice, and had reduced expression of IFN-β and CXCL10 [30,32]. Collectively, these reports provide evidence for the opposing effects of the deubiquitinase activity of OTUD3 on signaling pathways mediated by RLRs and cGAS: OTUD3 negatively regulates RLR-mediated type I IFN responses, whereas cGAS-mediated type I IFN responses are augmented by OTUD3 activation. Finally, it is worth noting that STING activation is negatively regulated by OTUD3. A recent study conducted by Li et al. showed that OTUD3 removes the K27-linked polyubiquitination of STING, thereby reducing type I IFN responses [17]. Thus, these studies clearly show that OTUD3 has dual roles in type I IFN responses; OTUD3 suppresses type I IFN responses mediated by RLRs through cleavage of K63-linked polyubiquitin chains on RIG-I, MDA5, and MAVS whereas cGAS-mediated type I IFN responses are augmented by OTUD3 through cleavage of K48-linked polyubiquitin chains on cGAS. However, the effects of OTUD3 on cGAS/STING-mediated type I IFN responses are complicated because this deubiquitinase also removes K27-linked polyubiquitin chains on STING and thereby suppresses type I IFN responses (Table 1).

The K63-linked polyubiquitination of TRAF3 is an essential process for TLR- and RLR-mediated type I IFN responses [16,27,28]. OTUD5 selectively cleaves K63-linked polyubiquitin chains on TRAF3 and inhibits the interaction between TRAF3 and TBK1 [33]. The dissociation of TRAF3 from the TBK1-containing signaling complex suppresses type I IFN production by macrophages and DCs. The siRNA-mediated knockdown of *Otud5* markedly increased expression of IFN-α upon activation with TLR3, TLR7, and RIG-I ligands [33]. Thus, OTUD5 functions as a negative regulator of type I IFN responses mediated by TLRs and RLRs. In contrast, OTUD5 promotes type I IFN responses mediated by the cGAS-STING signaling pathway. The removal of the K48-linked polyubiquitin chains from STING by OTUD5 enhances type I IFN production by macrophages by promoting STING protein stability [34]. Indeed, mice with myeloid lineage cell-specific deficiency in OTUD5 displayed increased sensitivity to herpes simplex virus infection due to the downregulation of type I IFN responses [34]. These findings suggest opposing effects of OTUD5 deubiquitinase activity on the signaling pathways mediated by TLRs/RLRs and cGAS: OTUD5 negatively regulates TLR/RLR-mediated type I IFN responses, whereas cGAS-mediated type I IFN responses are augmented by the activation of OTUD5. As in the case of OTUD3, OTUD5 has dual roles on type I IFN responses; OTUD5 suppresses type I IFN responses mediated by RLRs and TLRs through cleavage of K63-linked polyubiquitin chains on TRAF3 whereas cGAS/STING-mediated type I IFN responses are augmented by OTUD5 through cleavage of K48-linked polyubiquitin chains on STING (Table 1).

## 5. Roles Played by OTUD3 in the Development of Ulcerative Colitis

SNPs in *Otud3* (rs758098056: G > A) are linked to the development of human UC [17]. Although this variant results in the replacement of arginine with glutamine at amino acid position 90 (R90Q), the molecular mechanisms underlying the development of UC in the presence of the *Otud3* R90Q SNP are poorly understood. To elucidate the immunopathogenesis underlying the development of UC in the presence of this *Otud3* SNP, Li et al. used OTUD3-deficient mice and found that they developed more severe DSS-induced colitis than wild-type mice. They then created four types of conditional knockout mice, in which the mouse *Otud3* gene was knocked out selectively in CD4^+^ T cells, myeloid cells, epithelial cells, or fibroblasts, to identify the types of cells expressing OTUD3 that play colitogenic roles. Unexpectedly, mice with fibroblast-specific OTUD3 deficiency, created by crossing *Pdgfra-cre* and *Otud3^flox/flox^* mice developed severe DSS-induced colitis. Thus, the more severe disease course has been attributed to OTUD3 deficiency in colonic fibroblasts [17].

**Figure 2:**
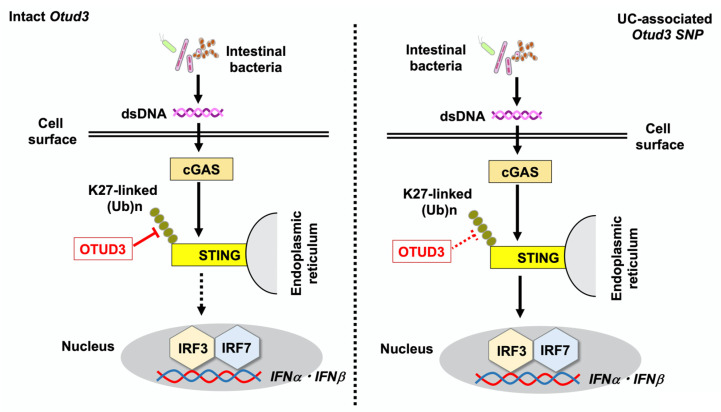
*Otud3* single nucleotide polymorphism (*Otud3* R90Q) is associated with the development of ulcerative colitis (UC). Double-stranded DNA (dsDNA) derived from intestinal bacteria is sensed by intracellular cyclic GMP-AMP synthase (cGAS), followed by the activation of the stimulator of interferon genes (STING), which induces the nuclear translocation of interferon regulatory factor 3 (IRF3) and 7 (IRF7) to initiate type I IFN responses. Intact OTUD3 expressed in fibroblasts downregulates cGAS-mediated type I IFN responses through the cleavage of K27-linked polyubiquitination of STING (**left panel**). In the presence of the *Otud3* R90Q polymorphism, impaired cleavage of polyubiquitin chains enhances type I IFN responses, causing UC (**right panel**).

Single-cell RNA sequencing studies revealed colonic accumulation of Ly6C^high^ platelet-derived growth factor receptor α (PDGFRα)^+^ fibroblasts deficient in the *Pdgfra-cre*; *Otud3^flox/flox^* mice. Accumulated Ly6C^high^ PDGFRα^+^ fibroblasts showed increased mRNA expression of type I IFN gene signature, including *Ifnα*, *Ifnβ*, *Irf3*, *Cgas*, *Sting*, and *signal transducer and activator of transcription 2* (*Stat2*), suggesting the involvement of excessive activation of cGAS-STING-mediated type I IFN responses in colonic fibroblasts (Figure 2) [17]. Indeed, colonic fibroblasts isolated from OTUD3-deficient mice produced large amounts of IFN-β upon stimulation with cGAS ligands as compared with those in wild-type mice. Importantly, the development of DSS-induced colitis was markedly attenuated in mice double deficient in STING and OTUD3. Therefore, these data suggest that OTUD3 deficiency exacerbated DSS-induced colitis through the excessive activation of type I IFN responses mediated by the cGAS-STING axis. To ascertain the clinical relevance of the experimental data obtained using OTUD3-deficient mice, the authors created *Otud3*^R89Q/R89Q^ mice in which the *Otud3* gene had a knock-in mutation (SNP R89Q) corresponding to the human UC *Otud3* SNP (R90Q). *Otud3*^R89Q/R89Q^ mice also developed more severe DSS-induced colitis [17]. Collectively, this study utilizing mice with fibroblast-specific OTUD3 deficiency or with UC-associated *Otud3* SNP knock-in provided evidence that cGAS-STING-mediated type I IFN responses exacerbate the development of DSS-induced colitis in the absence of OTUD3 or presence of UC-associated *Otud3* SNP.

Regarding the molecular mechanisms by which the UC-associated *Otud3* SNP enhances type I IFN responses, colonic fibroblasts isolated from *Otud3*^R89Q/R89Q^ mice and UC patients carrying heterozygous *Otud3*^R90Q^ displayed enhanced K27-linked polyubiquitination of STING compared to that in cells with intact OTUD3, resulting in the accumulation of colonic fibroblasts expressing higher levels of *IFNβ* and *CXCL10* upon stimulation with cGAS ligands [17]. In summary, impaired OTUD3 function in the presence of UC-associated *Otud3* SNP or the absence of *Otud3* enhances sensitivity to colonic inflammation through enhanced type I IFN responses mediated by K27-linked polyubiquitination of STING.

Excessive production of cytokines upon the recognition of intestinal bacteria by PRRs underlies the development of CD and UC [1,2,3]. Li et al. found that UC-associated microbiota was enriched in bacteria that could activate the cGAS-STING pathway [17]. Fecal microbiota transplantation experiments were conducted in four combinations: wild-type or *Otud3*^R89Q/R89Q^ mice transplanted with fecal bacteria from either healthy human controls or patients with UC. Interestingly, the degree of DSS-induced colitis was most severe in the *Otud3*^R89Q/R89Q^ mice transplanted with intestinal bacteria from patients with UC. Severe DSS-induced colitis seen in this condition was characterized by enhanced expression of type I IFN signature genes, IL-6 and TNF-α. Taken together, the experiments conducted by Li et al. successfully elucidated the immunopathogenesis of UC in the presence of *Otud3* SNP by providing evidence that OTUD3 protects against colitis by negatively regulating type I IFN responses. The frequency and clinical characteristics of UC patients carrying *Otud3* SNP have been poorly defined. The relationship between *Otud3* SNP and type I IFN responses needs to be clarified in future studies.

## 6. Roles of OTUD5 in the Development of IBD

Loss-of-function mutations in the caspase recruitment domain 15 (*Card15*) gene encoding nucleotide-binding oligomerization domain 2 (NOD2) are the strongest risk factor for CD [3,35]. NOD2 detects muramyl dipeptide (MDP) derived from intestinal bacteria and functions as an intracellular sensor in innate immunity [3,35]. Several mechanisms have been proposed as to the immunopathogenesis underlying the development of CD in the presence of *Card15* mutations [3,35]. Among these mechanisms, the crosstalk between TLRs and NOD2 partially explains the molecular mechanisms by which *Card15* mutations predispose to CD. Sensing of MDP by NOD2 negatively regulates production of TLR-mediated NF-κB-dependent proinflammatory cytokines such as IL-12, IL-23, and TNF-α in macrophages and DCs [3,36]. Thus, activation of NOD2 by MDP contributes to the maintenance of intestinal immune homeostasis through the inhibition of excessive production of IL-12, IL-23, and TNF-α by macrophages and DCs triggered by TLRs. In contrast, loss-of-function *Card15* mutations associated with CD enhance colitogenic cytokine responses upon exposure to TLR ligands derived from intestinal bacteria. Given that intestinal bacteria are enriched in NOD2 and TLR ligands, this dual recognition system for bacterial antigens prevents intestinal inflammation. Although the effects of the NOD2–TLR crosstalk on NF-κB-dependent cytokine responses have been clarified, how this crosstalk modulates type I IFN responses has not been properly defined.

**Figure 3:**
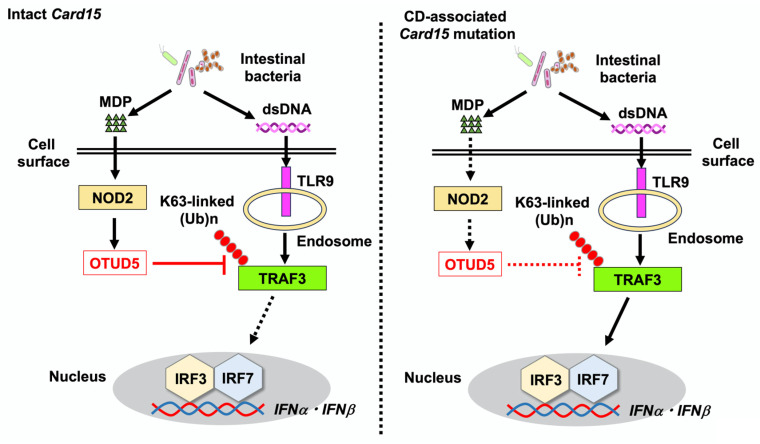
Loss-of-function mutations in caspase recruitment domain 15 (*Card15*), which encodes nucleotide-binding oligomerization domain 2 (NOD2), are associated with the development of Crohn’s disease (CD). NOD2 detects muramyl dipeptide (MDP) derived from intestinal bacteria. The recognition of double-stranded DNA (dsDNA) derived from intestinal bacteria by Toll-like receptor 9 (TLR9) induces strong type I IFN responses through the K63-linked polyubiquitination of TNF receptor-associated factor 3 (TRAF3) and nuclear translocation of interferon regulatory factor 3 (IRF3) and IRF7. Intact NOD2 expressed in macrophages and dendritic cells acts in concert with TLR9 to induce the expression of OTUD5, which removes the K63-linked polyubiquitination of TRAF3. TLR9-mediated type I IFN responses are suppressed by MDP activation of NOD2 through the deubiquitination of TRAF3 by OTUD5. In the presence of loss-of-function mutations in *Card15* associated with CD, the induction of OTUD5 expression is impaired. As a result, TLR9 activation causes colitogenic cytokine responses.

To address the effects of NOD2 activation on TLR-mediated type I IFN responses, human peripheral blood monocytes and pDCs were stimulated with MDP and CpG [18]. TLR9-mediated production of IFN-α and CXCL10 by human monocytes and pDCs was markedly reduced upon co-stimulation of NOD2 and TLR9 through the downregulation of transactivation of IRF3 and IRF7. Co-stimulation with NOD2 and TLR9 enhanced the expression of OTUD5, which removes polyubiquitin chains from TRAF3. Indeed, the K63-linked polyubiquitination of TRAF3 was markedly reduced in BM-derived DCs upon co-stimulation with MDP and CpG as compared with the effect of CpG stimulation alone. The siRNA-mediated knockdown of *Otud5* cancelled the NOD2-mediated negative regulation of IFN-α production triggered by TLR9 [18]. These in vitro experiments using human and murine macrophages and DCs suggest that MDP activation of NOD2 negatively regulates TLR9-mediated type I IFN responses by inhibiting the K63-linked polyubiquitination of TRAF3 (Figure 3).

CpG activation of TLR9 during the induction phase of DSS-induced colitis exacerbates colonic inflammation [11,12,13,18]. TLR9-induced exacerbation of colitis was type I IFN-dependent because colitis in mice lacking the type I IFN receptor was not modified by CpG injection [18]. Importantly, the exacerbation of DSS-induced colitis caused by TLR9 activation was not observed in mice that were intraperitoneally co-injected with MDP and CpG. Suppression of DSS-induced colitis by NOD2 activation was accompanied by reduced production of IFN-α and CXCL10 by colonic lamina propria mononuclear cells upon in vitro stimulation with lipopolysaccharide and CpG. Furthermore, hematopoietic cells expressing NOD2 were required to suppress colitis: irradiated wild-type mice transplanted with BM cells derived from NOD2-deficient mice developed severe colitis despite injection of MDP, whereas colitis development was markedly suppressed in irradiated NOD2-deficient mice transplanted with BM cells from mice with intact NOD2 [18]. Regarding the molecular mechanisms underlying the suppression of DSS- and CpG-induced severe colitis by MDP activation of NOD2, OTUD5 expression was markedly increased in the colons of mice treated with MDP and CpG. The siRNA-mediated knockdown of *Otud5* resulted in the development of severe DSS-induced colitis even with MDP activation of NOD2 through the upregulation of colonic expression of IFN-α and CXCL10. Collectively, these in vitro and in vivo studies strongly suggest that MDP activation of NOD2 inhibits TLR9-induced type I IFN responses through the removal of the K63-linked polyubiquitination of TRAF3 by OTUD5 activation, and thereby suppresses the development of TLR9-induced colitis.

These experimental findings are corroborated by immunofluorescence analyses of human IBD samples. The percentages of colonic lamina propria mononuclear cells expressing OTUD5 inversely correlated with those of cells expressing IFN-α in the colonic mucosa of patients with CD, but not UC [18]. *Otud5* and *Cxcl10* mRNA expression levels in the colonic mucosa were significantly higher and lower, respectively, in patients with CD in the remission phase than in those in the active phase [18]. The colonic mucosa of patients with UC in the active stage was characterized by enhanced mRNA expression levels of *Cxcl10*, *Tlr9*, and *Irf7* compared to those in patients in the remission phase. However, no significant alterations in *Otud5* mRNA expression were observed in the colonic mucosa of patients with UC between the active and remission phases. Collectively, studies conducted by Masuta et al. provided another mechanism underlying the development of CD in the presence of *Card15* mutations. Sensing of intestinal bacteria-derived MDP by intact NOD2 induces OTUD5 expression in colonic macrophages and DCs and then negatively regulates type I IFN responses triggered by TLR9 recognition of bacterial dsDNA through the deubiquitinase activity of OTUD5 against TRAF3. CD-associated *Card15* mutations are loss-of-function mutations that prevent the recognition of MDP [3,35]. In the presence of CD-associated *Card15* mutations, the impaired induction of OTUD5 expression is likely to increase TLR9-mediated type I IFN responses, leading to the development of CD. CD patients carrying *Card15* mutations exhibit severe phenotypes including stricture and ileal involvement, as compared with *Card15*-intact patients [37]. It would be intriguing to examine whether CD patients carrying *Card15* mutations display enhanced type I IFN responses due to reduced expression of OTUD5.

## 7. Conclusions and Future Directions

OTUD3 and OTUD5 regulate type I IFN responses triggered by PRRs, including RLRs, TLRs, and cGAS. Depending on the types of polyubiquitin chains cleaved by OTUD3 and OTUD5, type I IFN responses are negatively and positively regulated (Table 1). OTUD3 promotes and suppresses type I IFN responses through the removal of K48-linked polyubiquitin chains and K63-linked polyubiquitin chains on cGAS and RIG-I/MDA5/MAVS, respectively. Similarly, type I IFN responses are attenuated and augmented by removal of K63-linked polyubiquitin chains and K48-linked polyubiquitin chains on TRAF3 and STING by OTUD5, respectively. The blockade of type I IFN responses without impairing antiviral defenses may be difficult. However, such opposing effects on type I IFN responses lead us to propose that regulation of pathogenic type I IFN responses without affecting antiviral immune responses may be possible through pharmacological modulation of OTUD3 and OTUD5 activity. Elucidation of molecular mechanisms underlying such opposing effects on type I IFN responses by OTUD3 and OTUD5 and the identification of key molecules linked to both OTUDs may open up a new avenue for the development of novel treatments in patients with immune disorders including IBD.

Recent studies have identified an association between the impaired function of OTUD3 and OTUD5 and the development of IBD associated with genetic abnormalities. The UC-associated *Otud3* SNP increases the susceptibility to colitis through cGAS-STING-mediated type I IFN responses due to enhanced K27-linked polyubiquitination of STING [17]. Additionally, in CD associated with *Card15* mutations, enhanced K63-linked polyubiquitination of TRAF3 triggers the excessive production of type I IFNs [18]. These studies support the pathogenic rather than protective roles of type I IFNs in the development of IBD associated with genetic abnormalities. Validation studies addressing the frequency of *Otud3* and *Otud5* SNP in large IBD patient cohorts may strengthen the pathogenicity of type I IFNs in IBD. Most of the studies addressing the pathogenicity of type I IFNs utilized DSS-induced colitis because this model faithfully reflects characteristic findings of human IBD [17,18]. However, verification of the pathogenicity of type I IFNs requires other experimental colitis models such as trinitrobenzene sulfonic acid-induced colitis and IL-10-deficient mice.

Given that the colonic mucosa of patients with IBD is characterized by type I IFN gene signature even with intact *Card15* or *Otud3* [14,15,18], the blockade of type I IFN responses might be an alternative treatment option for patients with IBD. Pharmacological inhibition of STING and activation of OTUD5 may be good candidates for regulating the type I IFN responses associated with IBD. Regulation of gut microbiome has been considered as a target of intervention in IBD [38,39]. Gut colonization by artificially engineered bacteria that produce large amounts of MDP and low levels of cGAMP may be promising for reducing pathogenic type I IFN responses. Thus, it would be important to visualize the interplay between microbiota and type I IFN responses in IBD. Further studies addressing the pathogenicity of type I IFNs in IBD in relation to genetic abnormalities are required to develop novel treatments targeting type I IFN signaling pathways.

## Figures and Tables

**Table 1:** Regulation of type I IFN responses by OTUD3 and OTUD5 through deubiquitination.

	Target Protein	Type of Ubiquitin Chains Cleaved by OTUD3 or OTUD5	Type I IFN Responses [References]	Association with IBD
OTUD3	RIG-I	K63	Suppression [30]	
	MDA5	K63	Suppression [30]	
	MAVS	K63	Suppression [31]	
	cGAS	K27, K48	Enhancement [30,32]	
	STING	K27	Suppression [17]	UC
OTUD5	TRAF3	K63	Suppression [18,33]	CD
	STING	K48	Enhancement [34]	

CD: Crohn’s disease; cGAS: cyclic GMP-AMP synthase; IBD: inflammatory bowel diseases; MAVS: mitochondrial antiviral-signaling protein; MDA5: melanoma differentiation-associated protein 5; RIG-I: retinoic acid-inducible gene-I; STING: stimulator of interferon genes, TRAF3: TNF receptor-associated factor 3; UC: ulcerative colitis.

## Data Availability

The data presented in this study are available on request from the corresponding author.

## Abbreviations

The following abbreviations are used in this manuscript:

BM	Bone marrow
CARD15	Caspase recruitment domain 15
CD	Crohn’s disease
cGAMP	2′,3′-Cyclic GMP-AMP
cGAS	Cyclic GMP-AMP synthase
CXCL10	C-X-C motif chemokine ligand 10
DC	Dendritic cell
DSS	Dextran sodium sulfate
GI	Gastrointestinal
IBD	Inflammatory bowel diseases
IFN	Interferon
IRF	Interferon regulatory factor
MAVS	Mitochondrial antiviral-signaling protein
MDA5	Melanoma differentiation-associated protein 5
MDP	Muramyl dipeptide
MyD88	Myeloid differentiation factor 88
NF-κB	Nuclear factor-κB
NOD2	Nucleotide-binding oligomerization domain 2
OTUD	OTU deubiquitinase
pDC	Plasmacytoid dendritic cell
PDGFRα	Platelet-derived growth factor receptor Alpha
PRR	Pattern recognition receptor
RLR	Retinoic acid-inducible gene-I-like receptor
SNP	Single nucleotide polymorphism
STING	Stimulator of interferon genes
TBK1	TANK-binding kinase 1
TLR	Toll-like receptor
TRAF3	TNF receptor-associated factor 3
UC	Ulcerative colitis
